# Supplementary material for: Identification of the potential active site of the septal peptidoglycan polymerase FtsW
Source: PLoS Genet. 2022 Jan 5;18(1):e1009993. doi: 10.1371/journal.pgen.1009993 (PMC8765783; doi:10.1371/journal.pgen.1009993)
Supplement: S1 Text — (DOCX) [file pgen.1009993.s001.docx]

**Supplemental Information**

**Construction of strains and plasmids**

LYA4

The strain LYA4 (TB28, *zapA-mcherry cat<>frt*) was constructed by removal of the *cat* cassette using the FLP expressing plasmid pCP20 as described by Datsenko KA, *et al.*[1].

pDML2041 derivatives

Derivatives of pDML2041 (pETDuet, P_T7_::6Xhis-*ftsW,pbp3*) [2] harboring different alleles of *ftsW* were created by site-directed mutagenesis using the primer pairs listed in S3 Table.

pSEB429 derivatives

Derivatives of pSEB429 (pDSW208, P_204_::*ftsW*) harboring different alleles of *ftsW* were created by site-directed mutagenesis using the primer pairs listed in S3 Table.

pSD349 derivatives

Derivatives of pSD349 (pDSW210, P_206_::*ftsW-l60-gfp*) harboring different alleles of *ftsW* were created by site-directed mutagenesis using the primer pairs listed in S3 Table.

pLY103

The plasmid pLY103 (pBAD33, P*_BAD_*::*gfp-ftsN*) was constructed by ligation of an SphI/HindIII digested DNA fragment containing *gfp*-*ftsN* into pBAD33 digested with the same enzymes. The DNA fragment was amplified from plasmid pLY76 (pDSW209, P_206_::gfp-*ftsN*) using primers pLY103-N-F and pLY68-N-R.

pLY113

The plasmid pLY113 (pDSW207, P*_204_*::*gfp-linker-ftsI*) was constructed by ligation of an EcoRI/HindIII digested DNA fragment containing *gfp*-linker-*ftsI* into pDSW207 digested with the same enzymes. The DNA fragment was amplified from chromosome DNA from EC436 [MC4100, Δ(λattL-lom)::bla lacIq P_204_:*:gfp-ftsI*] using primers pLY113-gfp-F and pLY113-I-R.

pLY114

The plasmid pLY114 (pBAD33, P*_BAD_*::*gfp-linker-ftsI*) was constructed by ligation of an SphI/HindIII digested DNA fragment containing *gfp*-linker-*ftsI* into pBAD33 digested with the same enzymes. The *gfp*-linker-*ftsI* DNA fragment was obtained from pLY113 by PCR using primers pLY114-gfp-F and pLY113-I-R.

**References**:

1. Datsenko KA, Wanner BL. One-step inactivation of chromosomal genes in Escherichia coli K-12 using PCR products. Proc Natl Acad Sci U S A. 2000;97(12):6640-5.

2. Leclercq S, Derouaux A, Olatunji S, Fraipont C, Egan AJ, Vollmer W, et al. Interplay between Penicillin-binding proteins and SEDS proteins promotes bacterial cell wall synthesis. Sci Rep. 2017;7:43306.
