# Supplementary material for: Identification of the potential active site of the septal peptidoglycan polymerase FtsW
Source: PLoS Genet. 2022 Jan 5;18(1):e1009993. doi: 10.1371/journal.pgen.1009993 (PMC8765783; doi:10.1371/journal.pgen.1009993)
Supplement: S1 Table — (DOCX) [file pgen.1009993.s002.docx]

**Supplemental Information**

**S1 Table. Bacterial strains used in this study**

| Strain | Genotype | Source /Reference |
| --- | --- | --- |
| C43 (DE3) | *F – ompT hsdSB (rB- mB-) gal dcm (DE3)* | [1] |
| EC436 | MC4100, Δ(λattL-lom)::bla lacI^q^ P_204_:*:gfp-ftsI* | [2] |
| HC261 | TB28, *zapA-gfp cat* | ]3] |
| JS238 | MC1061 *malPp::lacIQ srlC::Tn10 recA1* | [4] |
| LYA4 | TB28, *zapA-mcherry cat<>frt* | This study |
| SD237 | W3110, *ftsW::kan* / pDSW406 | [5] |
| TU211 | TB28, *zapA-mcherry cat* | [3] |

**References**:

1. Miroux B, Walker JE. Over-production of proteins in Escherichia coli: mutant hosts that allow synthesis of some membrane proteins and globular proteins at high levels. J Mol Biol. 1996;260(3):289-98.

2. Mercer KL, Weiss DS. The Escherichia coli cell division protein FtsW is required to recruit its cognate transpeptidase, FtsI (PBP3), to the division site. J Bacteriol. 2002;184(4):904-12.

3. Peters NT, Dinh T, Bernhardt TG. A fail-safe mechanism in the septal ring assembly pathway generated by the sequential recruitment of cell separation amidases and their activators. J Bacteriol. 2011;193(18):4973-83.

4. Pichoff S, Vollrath B, Touriol C, Bouche JP. Deletion analysis of gene minE which encodes the topological specificity factor of cell division in Escherichia coli. Mol Microbiol. 1995;18(2):321-9.

5. Li Y, Gong H, Zhan R, Ouyang S, Park KT, Lutkenhaus J, et al. Genetic analysis of the septal peptidoglycan synthase FtsWI complex supports a conserved activation mechanism for SEDS-bPBP complexes. PLoS Genet. 2021;17(4):e1009366.
