# Supplementary material for: Identification of the potential active site of the septal peptidoglycan polymerase FtsW
Source: PLoS Genet. 2022 Jan 5;18(1):e1009993. doi: 10.1371/journal.pgen.1009993 (PMC8765783; doi:10.1371/journal.pgen.1009993)
Supplement: S2 Table — (DOCX) [file pgen.1009993.s003.docx]

**Supplemental Information**

**S2 Table. Plasmids used in this study**

| Plasmid | Genotype | Source /Reference |
| --- | --- | --- |
| pAB77 | pETDuet-*his-ftsN/pbp3* *bla* | [1] |
| pBAD33 | pACYC184*, cat* P_BAD_::*mcs* | [2] |
| pDML2040 | pETDuet-*his-pbp3/ftsW* *bla* | [3] |
| pDML2041 | pETDuet-*his-ftsW/pbp3 bla* | [3] |
| pDML2043 | pETDuet-*his-pbp3/ftsW_HA_* *bla* | [3] |
| pDSW207 | pDSW204, *bla* P_204_::*gfp-mcs* | [4] |
| pDSW208 | pDSW204, *bla* P_204_::*mcs-gfp* | [4] |
| pDSW209 | pDSW206, *bla* P_206_::*gfp-mcs* | [4] |
| pLY53 | pDSW208, *bla* P_204_::*ftsW^A135T^* | This study |
| pLY54 | pDSW208, *bla* P_204_::*ftsW^Q147E^* | This study |
| pLY55 | pDSW208, *bla* P_204_::*ftsW^P196T^* | This study |
| pLY56 | pDSW208, *bla* P_204_::*ftsW^G199A^* | This study |
| pLY57 | pDSW208, *bla* P_204_::*ftsW^Y242H^* | This study |
| pLY58 | pDSW208, *bla* P_204_::*ftsW^R243L^* | This study |
| pLY59 | pDSW208, *bla* P_204_::*ftsW^D297A^* | This study |
| pLY60 | pDSW208, *bla* P_204_::*ftsW^G371S^* | This study |
| pLY61 | pDSW208, *bla* P_204_::*ftsW^G381D^* | This study |
| pLY68 | pDSW208, *bla* P_204_::*ftsW^W138A^* | This study |
| pLY69 | pDSW208, *bla* P_204_::*ftsW^E150A^* | This study |
| pLY70 | pDSW208, *bla* P_204_::*ftsW^H295A^* | This study |
| pLY71 | pDSW208, *bla* P_204_::*ftsW^T296A^* | This study |
| pLY72 | pDSW208, *bla* P_204_::*ftsW^P368A^* | This study |
| pLY73 | pDSW208, *bla* P_204_::*ftsW^S378A^* | This study |
| pLY74 | pDSW208, *bla* P_204_::*ftsW^Y379A^* | This study |
| pLY75 | pDSW208, *bla* P_204_::*ftsW^G380A^* | This study |
| pLY86 | pDSW208, *bla* P_204_::*ftsW^E289G^* | [5] |
| pLY103 | pBAD33, *cat* P_BAD_::*gfp-ftsN* | This study |
| pLY113 | pDSW207, *bla* P_204_::*gfp-linker-ftsI* | This study |
| pLY114 | pBAD33, *cat* P_204_::*gfp-linker-ftsI* | This study |
| pLY295 | pDSW208, *bla* P_204_::*ftsW^A135T,E289G^* | This study |
| pLY296 | pDSW208, *bla* P_204_::*ftsW^Q147E,E289G^* | This study |
| pLY297 | pDSW208, *bla* P_204_::*ftsW^P196T,E289G^* | This study |
| pLY298 | pDSW208, *bla* P_204_::*ftsW^G199A,E289G^* | This study |
| pLY299 | pDSW208, *bla* P_204_::*ftsW^Y242H,E289G^* | This study |
| pLY300 | pDSW208, *bla* P_204_::*ftsW^R243L,E289G^* | This study |
| pLY301 | pDSW208, *bla* P_204_::*ftsW^E289G,G371S^* | This study |
| pLY302 | pDSW208, *bla* P_204_::*ftsW^E289G,G381D^* | This study |
| pLY303 | pDSW208, *bla* P_204_::*ftsW^W138A,E289G^* | This study |
| pLY304 | pDSW208, *bla* P_204_::*ftsW^E289G,Y379A^* | This study |
| pLY305 | pDSW208, *bla* P_204_::*ftsW^E289G,G380A^* | This study |
| pSD348 | pDSW210, *bla* P_206_::*l60-gfp* | [6] |
| pSD349 | pDSW210, *bla* P_206_::*ftsW-l60-gfp* | [5] |
| pSD349-A135T | pDSW210, *bla* P_206_::*ftsW^A135T^-l60-gfp* | This study |
| pSD349-Q147E | pDSW210, *bla* P_206_::*ftsW^Q147E^-l60-gfp* | This study |
| pSD349-P196T | pDSW210, *bla* P_206_::*ftsW^P196T^-l60-gfp* | This study |
| pSD349-G199A | pDSW210, *bla* P_206_::*ftsW^G199A^-l60-gfp* | This study |
| pSD349-Y242H | pDSW210, *bla* P_206_::*ftsW^Y242H^-l60-gfp* | This study |
| pSD349-R243L | pDSW210, *bla* P_206_::*ftsW^R243L^-l60-gfp* | This study |
| pSD349-G371S | pDSW210, *bla* P_206_::*ftsW^G371S^-l60-gfp* | This study |
| pSD349-G381D | pDSW210, *bla* P_206_::*ftsW^G381D^-l60-gfp* | This study |
| pSD349-R243L | pDSW210, *bla* P_206_::*ftsW^R243L^-l60-gfp* | This study |
| pSD349-G371S | pDSW210, *bla* P_206_::*ftsW^G371S^-l60-gfp* | This study |
| pSD349-G381D | pDSW210, *bla* P_206_::*ftsW^G381D^-l60-gfp* | This study |
| pSEB429 | pDSW208, *bla* P_204_::*ftsW* | [7] |
| pSEB429-DNM2 | pDSW208, *bla* P_204_::*ftsW^G199A^* | This study |
| pSEB429-DNM4 | pDSW208, *bla* P_204_::*ftsW^A135T^* | This study |
| pSEB429-DNM6 | pDSW208, *bla* P_204_::*ftsW^G371S^* | This study |
| pSEB429-DNM7 | pDSW208, *bla* P_204_::*ftsW^A135T^* | This study |
| pSEB429-DNM8 | pDSW208, *bla* P_204_::*ftsW^R243H^* | This study |
| pSEB429-DNM9 | pDSW208, *bla* P_204_::*ftsW^R246H^* | This study |
| pSEB429-DNM10 | pDSW208, *bla* P_204_::*ftsW^G381V,L214L,G227G,L264L^* | This study |
| pSEB429-DNM11 | pDSW208, *bla* P_204_::*ftsW^K133I,S130S^* | This study |
| pSEB429-DNM12 | pDSW208, *bla* P_204_::*ftsW^A135V^* | This study |
| pSEB429-DNM13 | pDSW208, *bla* P_204_::*ftsW^Q147K^* | This study |
| pSEB429-DNM14 | pDSW208, *bla* P_204_::*ftsW^R243S,A215A,I303I^* | This study |
| pSEB429-DNM15 | pDSW208, *bla* P_204_::*ftsW^G381D,G261G^* | This study |
| pSEB429-DNM16 | pDSW208, *bla* P_204_::*ftsW^R137H^* | This study |
| pSEB429-DNM17 | pDSW208, *bla* P_204_::*ftsW^L198I,R245L^* | This study |
| pSEB429-DNM18 | pDSW208, *bla* P_204_::*ftsW^G381V,L214L,G227G,L264L^* | This study |
| pSEB429-DNM19 | pDSW208, *bla* P_204_::*ftsW^Q147E^* | This study |
| pSEB429-DNM20 | pDSW208, *bla* P_204_::*ftsW^R243P,G117G^* | This study |
| pSEB429-DNM21 | pDSW208, *bla* P_204_::*ftsW^P196T^* | This study |
| pSEB429-DNM22 | pDSW208, *bla* P_204_::*ftsW^R243L,S130S^* | This study |
| pSEB429-DNM23 | pDSW208, *bla* P_204_::*ftsW^G199A,S136S,A232V^* | This study |
| pSEB429-DNM24 | pDSW208, *bla* P_204_::*ftsW^R246H,G183C^* | This study |
| pSEB429-DNM25 | pDSW208, *bla* P_204_::*ftsW^Y242H,F106Y^* | This study |
| pSEB429-R245L | pDSW208, *bla* P_204_::*ftsW^R245L^* | This study |
| pSEB429-L198I | pDSW208, *bla* P_204_::*ftsW^L198I^* | This study |
| pSEB429-A232V | pDSW208, *bla* P_204_::*ftsW^A232V^* | This study |
| pSEB429-G183C | pDSW208, *bla* P_204_::*ftsW^G183C^* | This study |
| pSEB429-Y242H | pDSW208, *bla* P_204_::*ftsW^Y242H^* | This study |
| pSEB429-F106Y | pDSW208, *bla* P_204_::*ftsW^F106Y^* | This study |

**References**:

1. Boes A. Étude des interactions et des régulations au sein du noyau de synthèse du peptidoglycane septal d’E. coli [Ph.D. Thesis] University of Liège; 2020. Available from: <http://hdl.handle.net/2268/254096>

2. Guzman LM, Belin D, Carson MJ, Beckwith J. Tight regulation, modulation, and high-level expression by vectors containing the arabinose PBAD promoter. J Bacteriol. 1995;177(14):4121-30.

3. Leclercq S, Derouaux A, Olatunji S, Fraipont C, Egan AJ, Vollmer W, et al. Interplay between Penicillin-binding proteins and SEDS proteins promotes bacterial cell wall synthesis. Sci Rep. 2017;7:43306.

4. Mercer KL, Weiss DS. The Escherichia coli cell division protein FtsW is required to recruit its cognate transpeptidase, FtsI (PBP3), to the division site. J Bacteriol. 2002;184(4):904-12.

5. Li Y, Gong H, Zhan R, Ouyang S, Park KT, Lutkenhaus J, et al. Genetic analysis of the septal peptidoglycan synthase FtsWI complex supports a conserved activation mechanism for SEDS-bPBP complexes. PLoS Genet. 2021;17(4):e1009366.

6. Du S, Lutkenhaus J. The N-succinyl-l,l-diaminopimelic acid desuccinylase DapE acts through ZapB to promote septum formation in Escherichia coli. Mol Microbiol. 2017;105(2):326-45.

7. Pichoff S, Du S, Lutkenhaus J. The bypass of ZipA by overexpression of FtsN requires a previously unknown conserved FtsN motif essential for FtsA-FtsN interaction supporting a model in which FtsA monomers recruit late cell division proteins to the Z ring. Mol Microbiol. 2015;95(6):971-87.
