# Supplementary material for: Identification of the potential active site of the septal peptidoglycan polymerase FtsW
Source: PLoS Genet. 2022 Jan 5;18(1):e1009993. doi: 10.1371/journal.pgen.1009993 (PMC8765783; doi:10.1371/journal.pgen.1009993)
Supplement: S3 Table — (DOCX) [file pgen.1009993.s004.docx]

**Supplemental Information**

**S3 Table. Primers used in this study**

| Primer name | Sequence |
| --- | --- |
| ftsW-F106Y-F | CGTCTGCCGATGGAGTACTGGCAACGCTACAGTG |
| ftsW-F106Y-R | CACTGTAGCGTTGCCAGTACTCCATCGGCAGACG |
| ftsW-A135T-F | GTAGCTCGGTTAAAGGGACATCGCGTTGGATCG |
| ftsW-A135T-R | CGATCCAACGCGATGTCCCTTTAACCGAGCTAC |
| ftsW-W138A-F | GTTAAAGGGGCATCGCGTGCGATCGATCTCGGTTTG |
| ftsW-W138A-R | CAAACCGAGATCGATCGCACGCGATGCCCCTTTAAC |
| ftsW-Q147E-F | CGGTTTGCTGCGTATCGAGCCTGCGGAGCTGAC |
| ftsW-Q147E-R | GTCAGCTCCGCAGGCTCGATACGCAGCAAACCG |
| ftsW-E150A-F | CGTATCCAGCCTGCGGCGCTGACAAAACTGTCG |
| ftsW-E150A-R | CGACAGTTTTGTCAGCGCCGCAGGCTGGATACG |
| ftsW-G183C-F | CTTCCTGAAACCGATGTGCGTGATTCTGGTGTTG |
| ftsW-G183C-R | CAACACCAGAATCACGCACATCGGTTTCAGGAAG |
| ftsW-P196T-F | GTTACTGCTGGCACAGACAGACCTTGGTACGGTG |
| ftsW-P196T-R | CACCGTACCAAGGTCTGTCTGTGCCAGCAGTAAC |
| ftsW-L198I-F | CTGGCACAGCCAGACATTGGTACGGTGGTGGTG |
| ftsW-L198I-R | CACCACCACCGTACCAATGTCTGGCTGTGCCAG |
| ftsW-G199A-F | GCACAGCCAGACCTTGCTACGGTGGTGGTGTTG |
| ftsW-G199A-R | CAACACCACCACCGTAGCAAGGTCTGGCTGTGC |
| ftsW-A232V-F | GGTATGGGCATTTCAGTGGTTGTGTTGCTGATAC |
| ftsW-A232V-R | GTATCAGCAACACAACCACTGAAATGCCCATACC |
| ftsW-Y242H-F | GATACTCGCCGAACCGCACCGTATCCGCCGTGTTAC |
| tsW-Y242H-R | GTAACACGGCGGATACGGTGCGGTTCGGCGAGTATC |
| ftsW-R243L-F | CTCGCCGAACCGTACCTTATCCGCCGTGTTACC |
| ftsW-R243L-R | GGTAACACGGCGGATAAGGTACGGTTCGGCGAG |
| ftsW-R245L-F | GAACCGTACCGTATCCTCCGTGTTACCGCATTC |
| ftsW-R245L-R | GAATGCGGTAACACGGAGGATACGGTACGGTTC |
| ftsW-R246H-F | CCGTACCGTATCCGCCATGTTACCGCATTCTGG |
| ftsW-R246H-R | CCAGAATGCGGTAACATGGCGGATACGGTACGG |
| ftsW-H295A-F | GTATCTGCCGGAAGCGGCCACTGACTTTATTTTC |
| ftsW-H295A-R | GAAAATAAAGTCAGTGGCCGCTTCCGGCAGATAC |
| ftsW-T296A-F | CTGCCGGAAGCGCACGCTGACTTTATTTTCGCC |
| ftsW-T296A-R | GGCGAAAATAAAGTCAGCGTGCGCTTCCGGCAG |
| ftsW-D297A-F | CCGGAAGCGCACACTGCCTTTATTTTCGCCATTATC |
| ftsW-D297A-R | GATAATGGCGAAAATAAAGGCAGTGTGCGCTTCCGG |
| ftsW-P368A-F | GCGGCGGGGATGTTAGCGACCAAAGGTCTGACATTG |
| ftsW-P368A-R | CAATGTCAGACCTTTGGTCGCTAACATCCCCGCCGC |
| ftsW-G371S-F | GATGTTACCGACCAAAAGTCTGACATTGCCGCTG |
| ftsW-G371S-R | CAGCGGCAATGTCAGACTTTTGGTCGGTAACATC |
| ftsW-S378A-F | GACATTGCCGCTGATCGCTTACGGTGGTTCGAGC |
| ftsW-S378A-R | GCTCGAACCACCGTAAGCGATCAGCGGCAATGTC |
| ftsW-Y379A-F | CATTGCCGCTGATCAGTGCCGGTGGTTCGAGCTTAC |
| ftsW-Y379A-R | GTAAGCTCGAACCACCGGCACTGATCAGCGGCAATG |
| ftsW-G380A-F | CCGCTGATCAGTTACGCTGGTTCGAGCTTACTG |
| ftsW-G380A-R | CAGTAAGCTCGAACCAGCGTAACTGATCAGCGG |
| ftsW-G381D-F | CTGATCAGTTACGGTGATTCGAGCTTACTGATTATG |
| ftsW-G381D-R | CATAATCAGTAAGCTCGAATCACCGTAACTGATCAG |
| pLY103-N-F | CGGCATGCTCACACAGGAAACAGACCAT |
| pLY68-N-R | GCAAGCTTTCAACCCCCGGCGGCGAGCC |
| pLY113-gfp-F | GGATGAACTATACAAAGAATTCAACAACAACAAAGCAG |
| pLY113-I-R  pLY114-gfp-F | ATCCGCCAAAACAGCCAAGCTTTTACGATCTGCCACCT  GAGTCGACCTGCAGGCATGCTCACACAGGAAACAGACC |
