# Supplementary figures and images for: Identification of the potential active site of the septal peptidoglycan polymerase FtsW
Source: PLoS Genet. 2022 Jan 5;18(1):e1009993. doi: 10.1371/journal.pgen.1009993 (PMC8765783; doi:10.1371/journal.pgen.1009993)

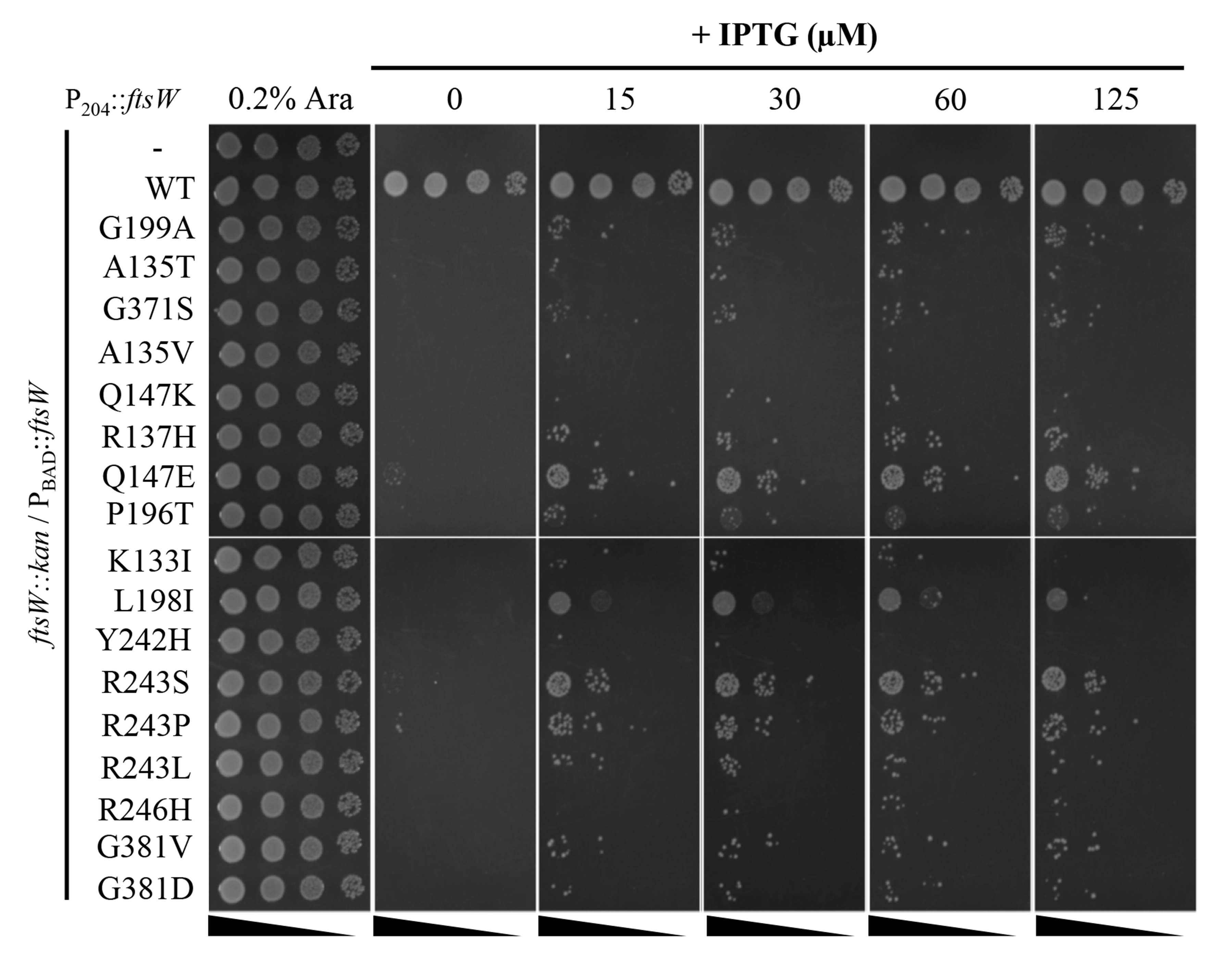

Supplement: S1 Fig — Plasmids pDSW208, pSEB429 (pDSW08, P204::ftsW) or its derivatives harboring a dominant-negative ftsW mutation were transformed into strain SD237 [W3110, leu::Tn10 ftsW::kan/pDSW406 (pBAD33, PBAD::ftsW)] on LB plates with ampicillin and 0.2% arabinose. The next day, a single transformant of each resulting strain was resuspended in 1 ml of LB medium, and serially diluted ten-fold. 3 μl of each dilution was spot on LB plates with antibiotics, with or without increasing concentrations of IPTG. Plates were incubated at 30°C overnight and imaged. (TIF) [file pgen.1009993.s005.tif]

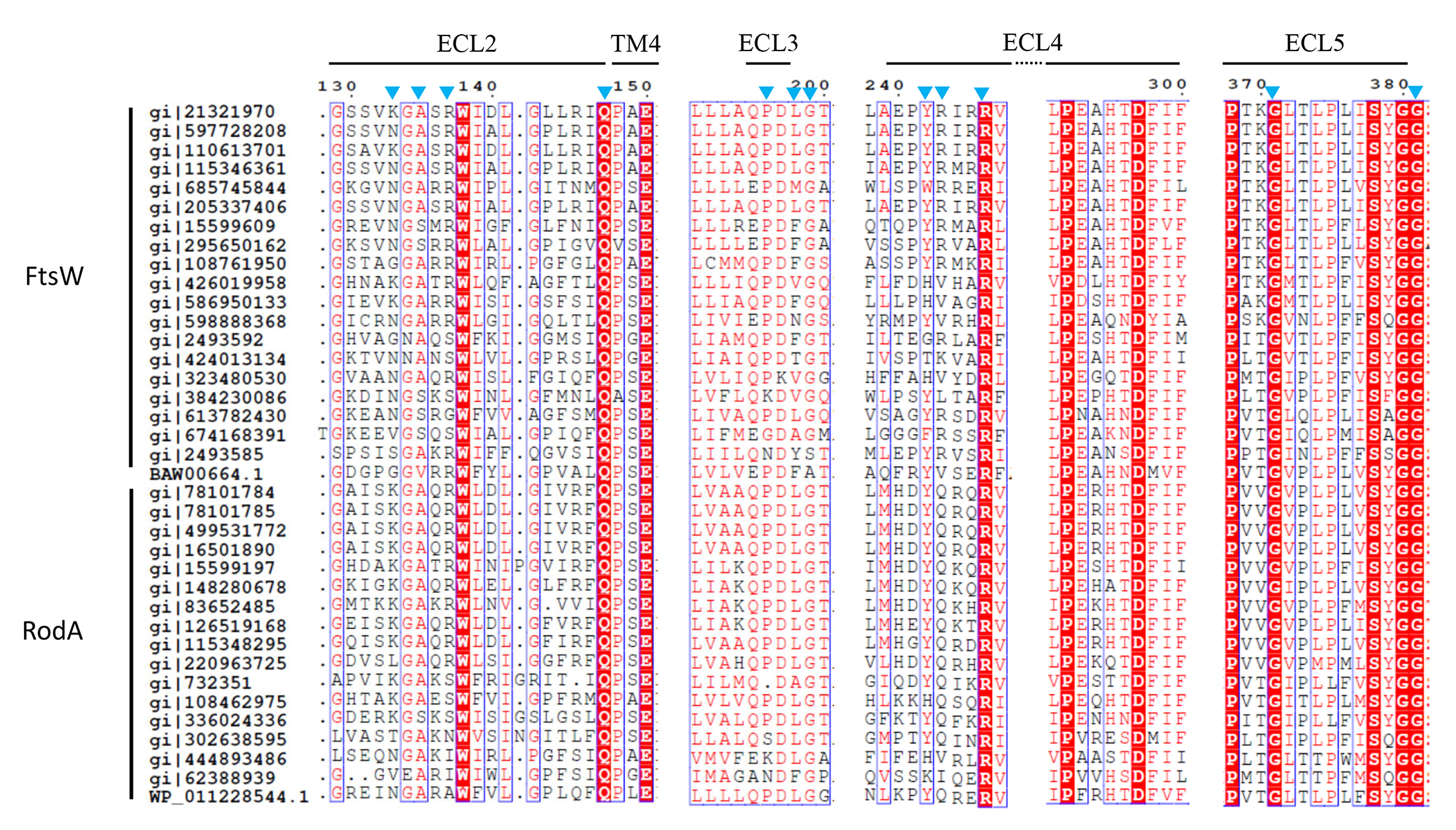

Supplement: S2 Fig — Amino acid sequences of FtsW and RodA were obtained from NCBI, aligned with Clustal Omega and then depicted using ESPRIPT: http://espript.ibcp.fr/. Residues were numbered according to the E. coli FtsW sequence and those corresponding to the dominant-negative mutations are indicated by blue triangles. FtsW: E. coli (gi|2132970), K. pneumoniae (gi|597728208), S. flexneri (gi|110613701), Y. pestis (gi|115346361), B. thailandensis (gi|685745844), S. enterica (gi|205337406), P. aeruginosa (gi|15599609), L. pneumophila (gi|295650162), M. xanthus (gi|108761950), C. crescentus (gi|426019958), A. tumefaciens (gi|586950133), B. fragilis (gi|598888368), B. subtilis (gi|2493592), L. monocytogenes (gi|424013134), E. faecalis (gi|323480530), S. aureus (gi|384230086), M. tuberculosis (gi|613782430), C. glutamicum (gi|674168391), B. burgdoferi (gi|2493585), T. thermophiles (BAW00664.1). RodA: E. coli (gi|78101784), S. flexneri (gi|78101785), K. pneumonia (gi|499531772), S. enterica (gi|16501890), P. aeruginosa (gi|15599197), L. pneumophila (gi|148280678), B. thailandensis (gi|83652485), V. cholera (gi|126519168), Y. pestis (gi|115348295), C. crescentus (gi|220963725), B. subtilis (gi|732351), M. xanthus (gi|108462975), L. monocytogenes (gi|336024336), S. pneumoniae (gi|302638595), M. tuberculosis (gi|444893486), C. glutamicum (gi|62388939), T. thermophilus (WP_011228544.1). (TIF) [file pgen.1009993.s006.tif]

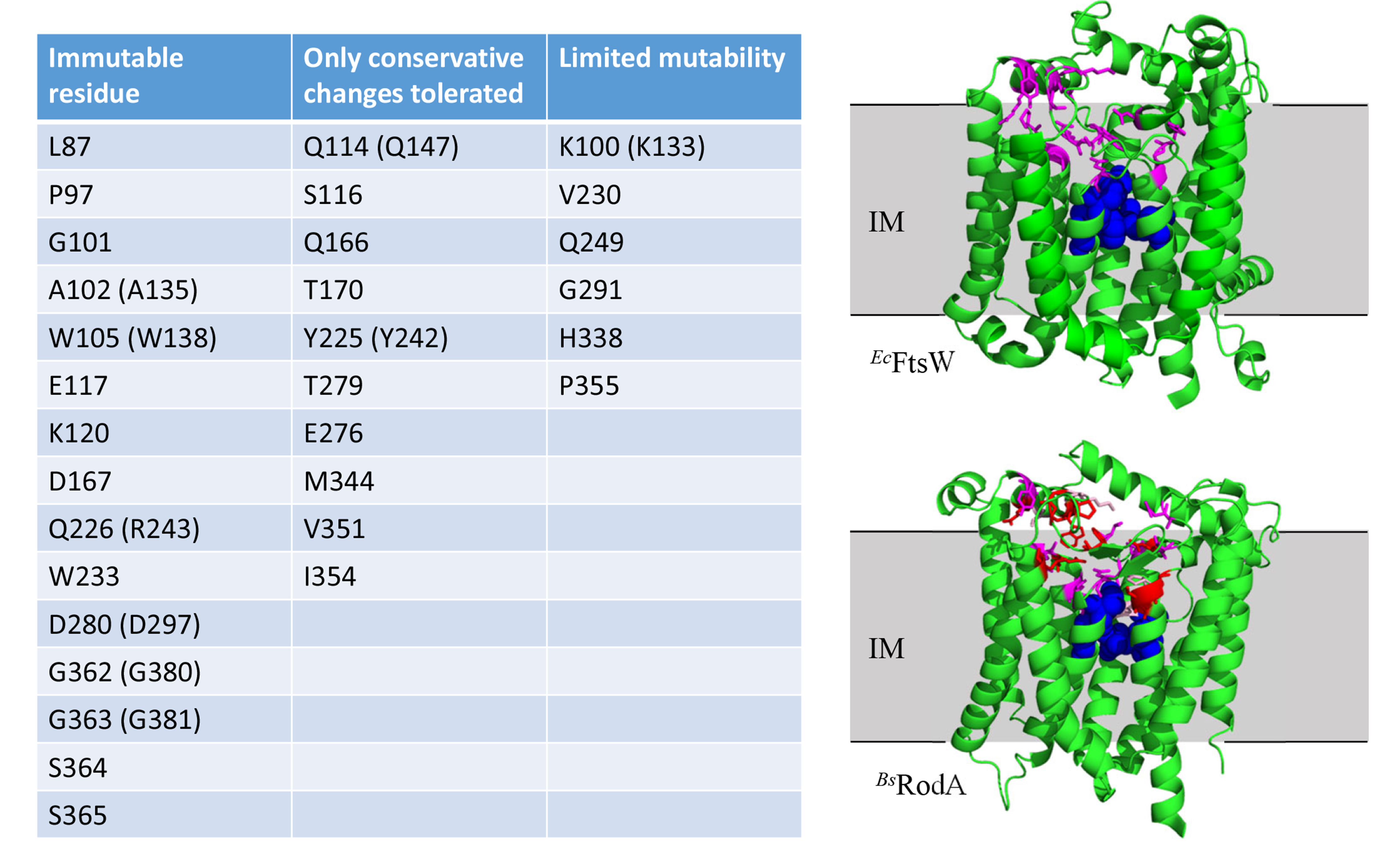

Supplement: S3 Fig — Mutability of critical residues of B. subtilis RodA were categorized based on previous Mutseq analysis [5]. Dominant-negative E. coli FtsW mutations isolated in this study that alter identical residues in B. subtilis RodA are indicated in parentheses. Residues critical for B. subtilis function are mapped to a model of RodA and the residues of FtsW whose substitutions displayed a dominant-negative effect are mapped to a model of FtsW. Red: immutable residues; magenta: residues that tolerate only conservative changes (dominant-negative mutations in FtsW); pink: residues with limited mutability. (TIF) [file pgen.1009993.s007.tif]

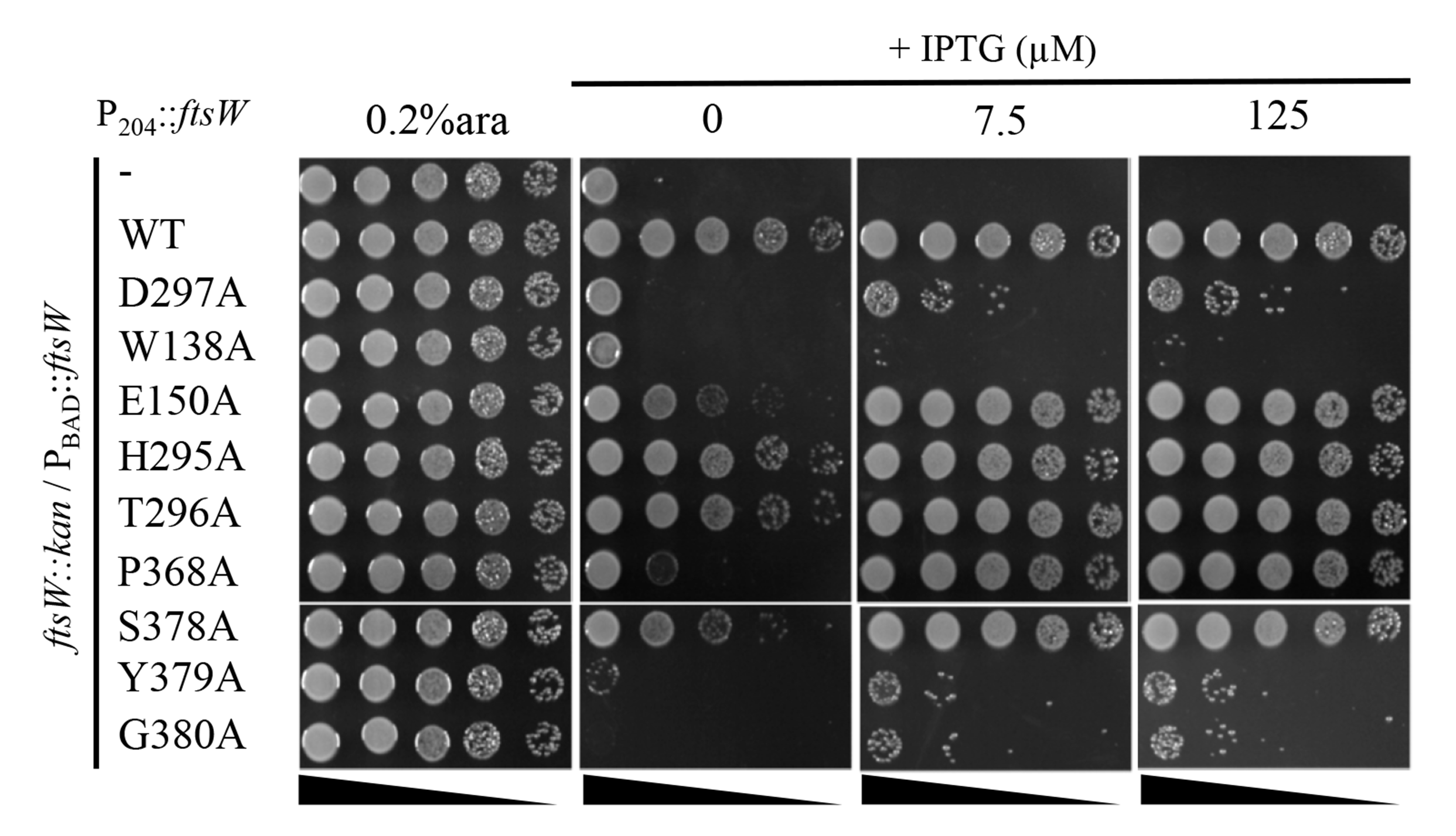

Supplement: S4 Fig — Transformation and spot tests were performed as in S1 Fig. (TIF) [file pgen.1009993.s008.tif]

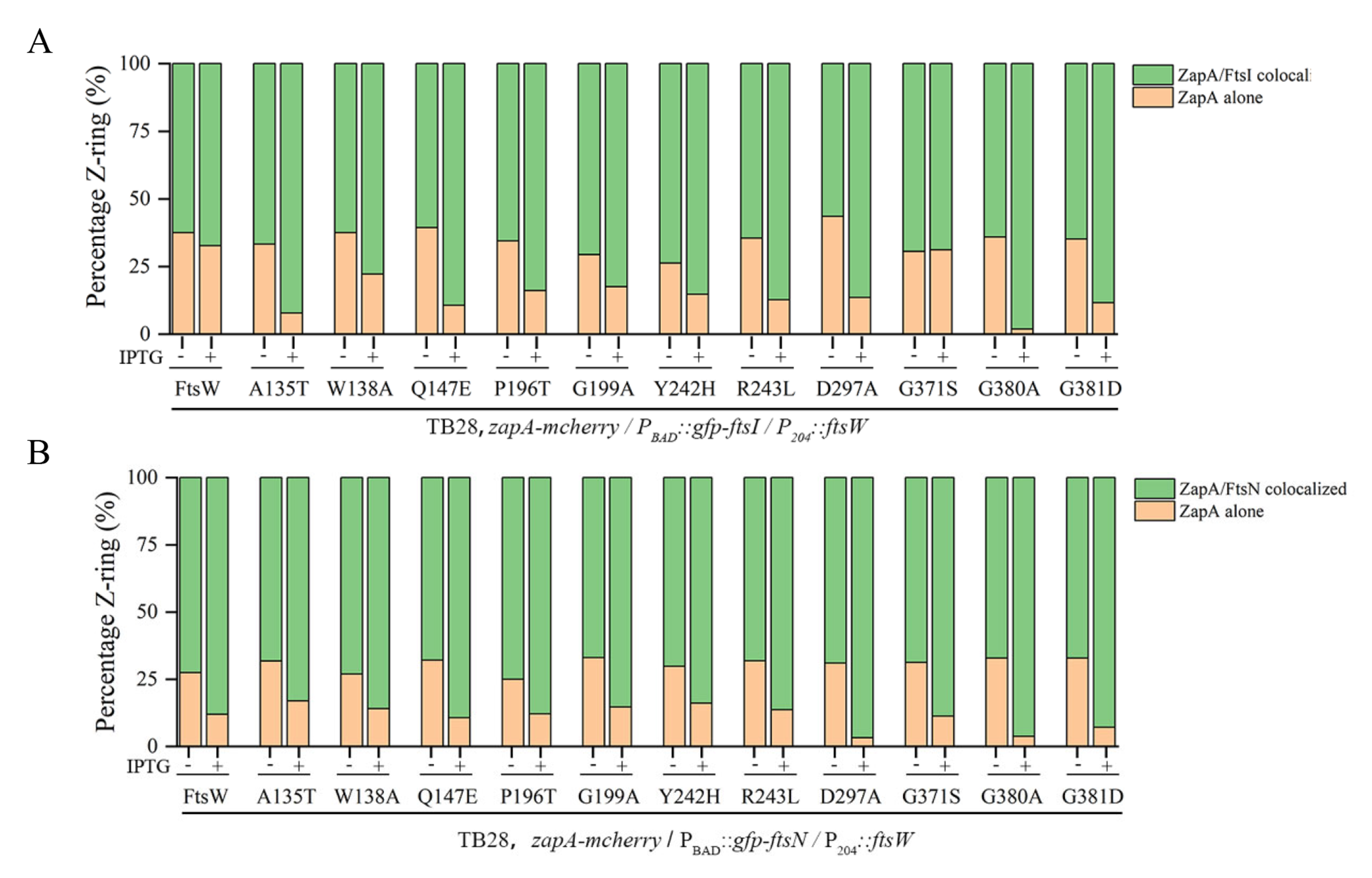

Supplement: S5 Fig — ZapA-mcherry rings and GFP-FtsI or GFP-FtsN rings displayed in Fig 6 and 7 were identified manually using ImageJ software. 50–200 ZapA-mcherry rings were examined for each strain and condition and the associated GFP-FtsI rings or GFP-FtsN rings were counted and plotted. (TIF) [file pgen.1009993.s009.tif]

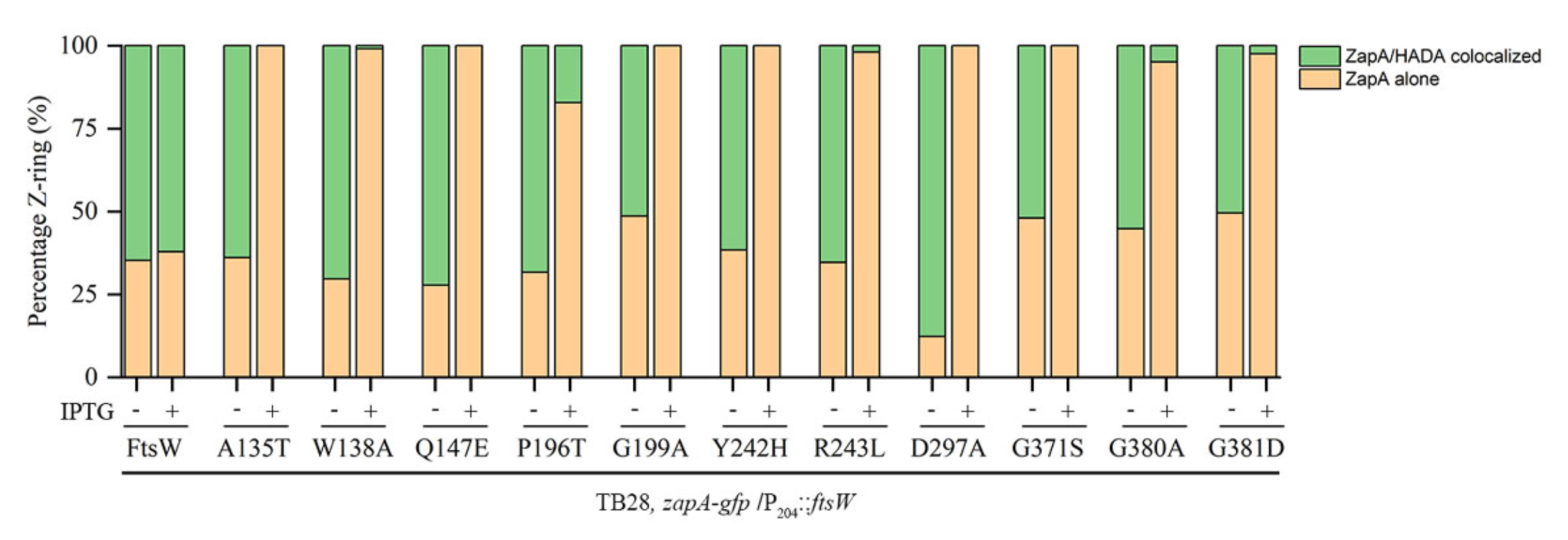

Supplement: S6 Fig — ZapA-mcherry rings and HADA bands represented in Fig 8 were identified manually using ImageJ software. 50–200 ZapA-mcherry rings were examined for each strain and condition and the associated HADA bands were counted and plotted. (TIF) [file pgen.1009993.s010.tif]

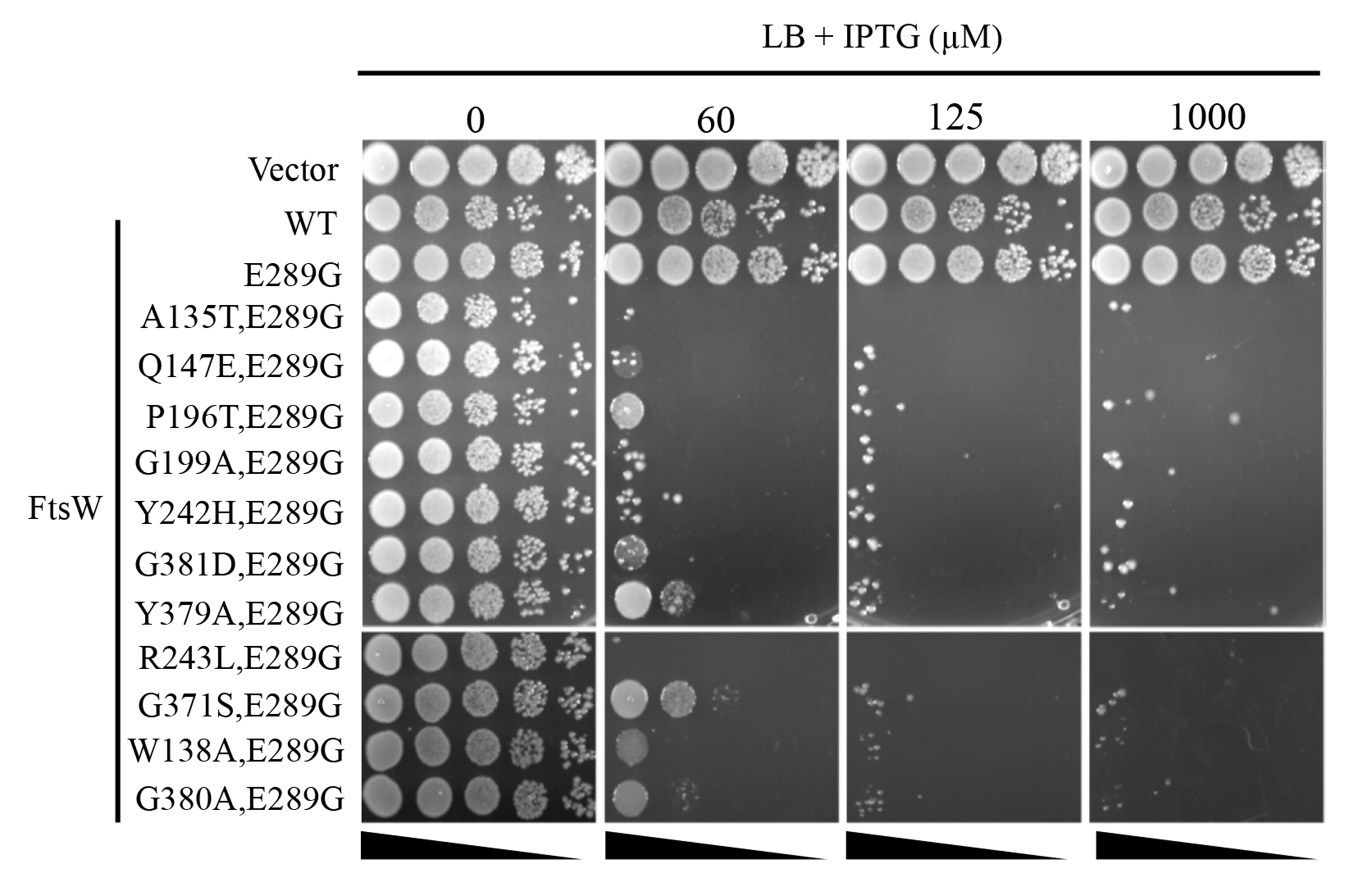

Supplement: S7 Fig — Transformation and spot test was performed as in Fig 1A. None of the dominant-negative ftsW mutations was suppressed by E289G. (TIF) [file pgen.1009993.s011.tif]

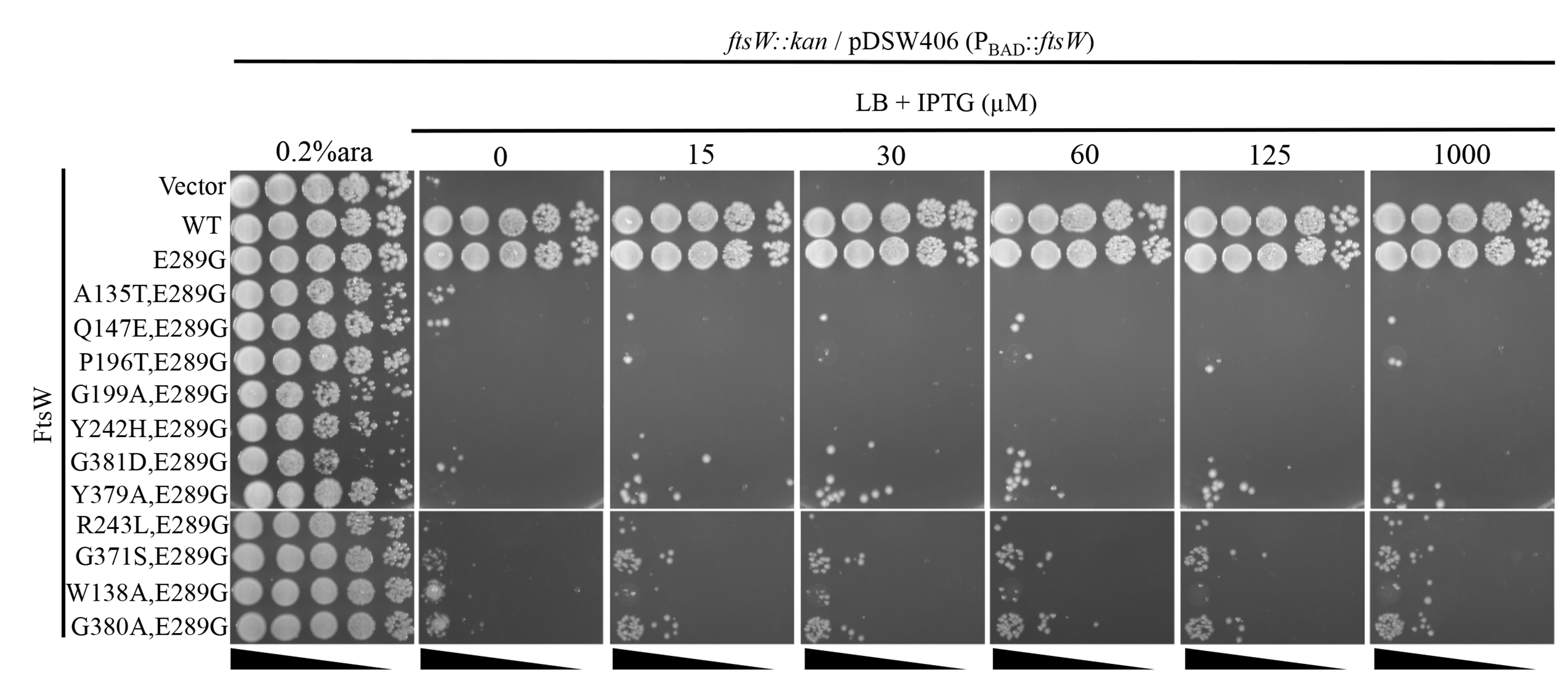

Supplement: S8 Fig — Transformation and spot test was performed as in S1 Fig. None of the dominant-negative ftsW mutations was suppressed by E289G. (TIF) [file pgen.1009993.s012.tif]

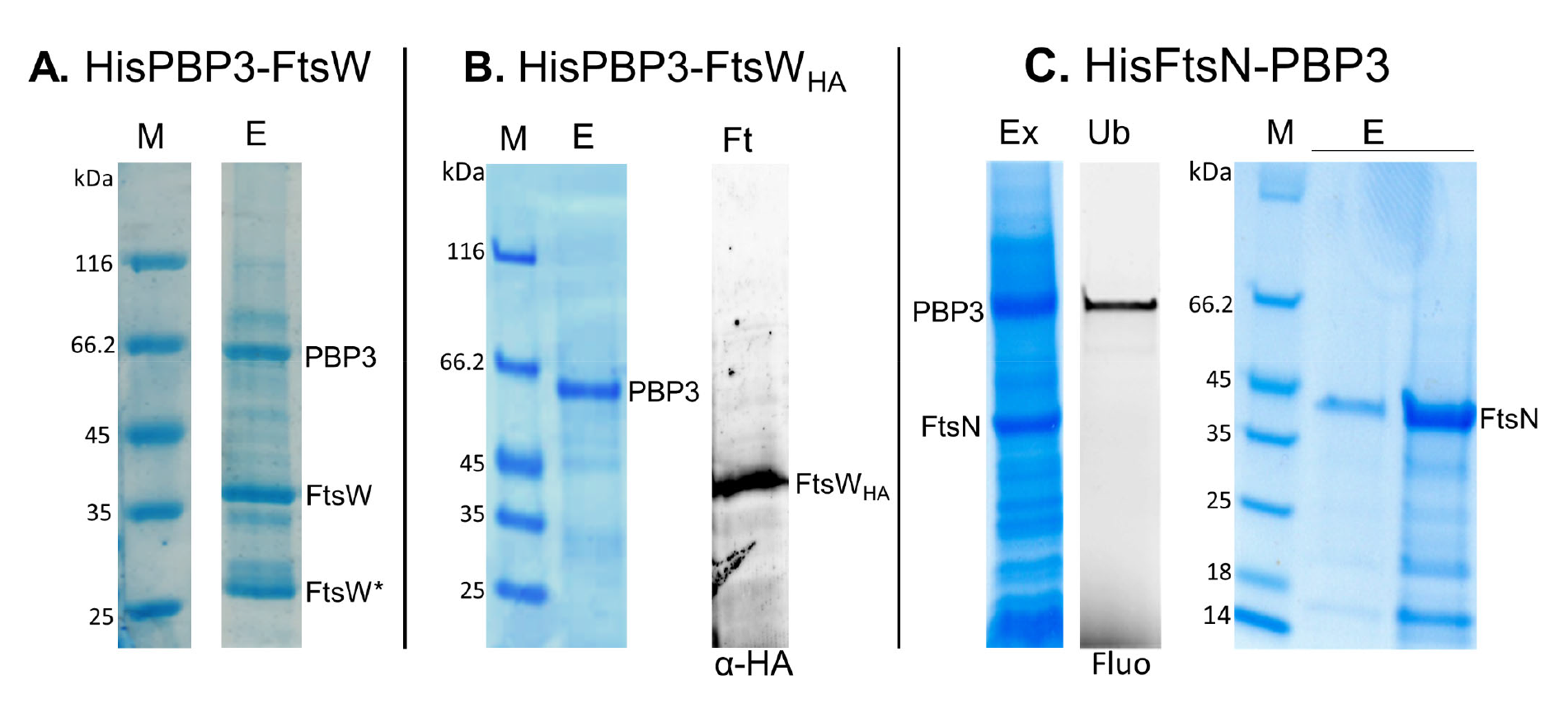

Supplement: S9 Fig — Protein-protein interaction was assessed by co-expression and co-purification of His-tagged protein with untagged protein as indicated above each panel. The proteins are co-expressed in E. coli and the membrane fractions isolated and solubilized by DDM detergent followed by purification on HisTrap column. The panels A-C show the SDS-PAGE analysis of the elution fractions. (A) Untagged FtsW co-elutes with His-tagged FtsI/PBP3. (B) Untagged FtsWHA, which does not bind to FtsI/PBP3, is not retained on a HisTrap column and was detected in the flow through using anti-HA antibodies. (C) When untagged FtsI/PBP3 was co-expressed with HisFtsN and purified in the same condition as HisFtsW-FtsI/PBP3, only HisFtsN was present in the elution fraction and PBP3 was detected in the unbound (Ub) fractions (detect by using Bocillin labelling (Fluo)), indicating no interaction between HisFtsN and PBP3. M, protein standard; E, elution fractions; Ft, flow through; FtsW* is a degradation product of FtsW. FtsWHA contains an insertion of a nine amino acid hemagglutinin (HA) peptide in the large loop between TM7 and TM 8. α-HA, immunoblot analysis using antibodies against the HA epitope of FtsWHA. Ex, membrane extraction fraction. Fluo, fluorescence analysis of PBP3 labeled with Bocillin. (TIF) [file pgen.1009993.s013.tif]
