# Supplementary material for: Identification of the potential active site of the septal peptidoglycan polymerase FtsW
Source: PLoS Genet. 2022 Jan 5;18(1):e1009993. doi: 10.1371/journal.pgen.1009993 (PMC8765783; doi:10.1371/journal.pgen.1009993)
Supplement: Attachment — Submitted filename: 12-7-2021 Response-to-reviewers.docx [file pgen.1009993.s014.docx]

**Responses to the reviewer’s comments**

**Reviewer #1:** In this manuscript by Li & Boes, the investigators present a genetic analysis of E. coli FtsW, a SEDS enzyme responsible for polymerization during division. FtsW works together with its enzymatic partner FtsI (also called PBP3) in the context of a multi-protein assembly, the divisome, to accomplish PG synthesis. While the polymerase activity of FtsW is well-established at this point, the molecular details of its enzymatic mechanism remain incompletely understood. Specifically, the mechanism by which the enzyme interacts with its lipid II substrate and accomplishes glycan chain polymerization is largely unknown. To identify residues involved in catalysis, the authors screen a library of FtsW mutants using a dominant negative assay based on a wrinkled colony phenotype. This strategy ensures that the identified mutations do not impair protein function at the level of expression or folding. Genetic analysis reveals a handful of mutants in the extracellular loops of FtsW as well as in the central “cavity” of the protein that inhibit division, consistent with previous reports on the homologous glycosyltransferase RodA. The authors suggest that nonfunctional mutants of FtsW preclude its catalytic activity rather than localization in the cell, since all of the identified mutants successfully localize to the division site but fail to produce PG in vivo. The authors then use a fluorescence anisotropy assay to probe lipid II binding to show that mutations do not affect lipid II binding, and so, the authors argue, must instead disrupt polymerization.

Overall, the isolation and validation of the mutants using in vivo assays is carefully performed and presented clearly. A major shortcoming of the manuscript is that the exact cause of the dominant negative effect of the mutations is not definitively established. Evidence is presented showing that localization, lipid binding, and downstream protein recruitment are intact in the mutants, leaving defects in catalysis as the most likely explanation. However, this is not directly assessed or demonstrated, and the authors state that they were unable to establish a polymerization assay for WT FtsW/FtsI complex. The result is that the data presented, while interesting, are incomplete for fully understanding what is happening with the mutants.

**Response**

We thank reviewer #1 for the comments and constructive suggestions to improve the manuscript. We also agree with the reviewer that it is critical to directly demonstrate that the FtsW mutants are disrupted for catalysis *in vitro* to fully understanding the defect of the mutants. However, in our hands the purified FtsW-FtsI complex from *E. coli* did not display any polymerase activity *in vitro*, which makes it impossible to us to probe the activity of the FtsW mutants. In fact, other labs also found that it is very difficult to obtain FtsW-FtsI from *E. coli* that are active for PG polymerization (Marmont and Bernhardt, Proc Natl Acad Sci U S A. 2020, 117(38):23879-23885). One way to confirm these FtsW mutants are defective in catalysis is to generate similar substitutions in FtsW from other organisms, such as *P. aeruginosa* which behaves well in PG polymerization assays *in vitro*, and repeat all the assays we had done with *E. coli* FtsW. However, this would take quite a while and the results are not guaranteed. Based on the *in vivo* and *in vitro* characterization of the mutants, we think it is reasonable to suggest that the residues corresponding to the dominant-negative mutations of FtsW constitute its potential active site. We tried to be as cautious as possible with our wordings in the revised manuscript to not exclude other possibilities.

**Main issues:**
1. In figures 5-8, the authors present imaging data to show localization of various divisome components to the septum. These representative images are compelling and consistent with the scientific conclusions of the manuscript. However, it would be useful to quantify the colocalization of the Z-ring with the proteins of interest (or HADA signal) and verify that the effect holds on a large dataset. This is particularly important in Figure 8, since the HADA signal appears variable in both the WT and FtsW mutant cells.

**Response**

We thank the reviewer for the suggestions. In the revised manuscript, we had quantified the co-localization of GFP-FtsI or GFP-FtsN with ZapA-mcherry as well as the co-localization of HADA labelling with ZapA-GPF. These results are consistent with the representative images in Figure 6, 7, and 8. Before induction of the FtsW variants, we found that about 70% of the ZapA-mcherry rings were associated with GFP-FtsI or GFP-FtsN rings as expected. After induction of the FtsW variants, the frequency of co-localization was increased, but there were also a few ZapA-mcherry rings without a GFP-FtsI or GFP-FtsN rings, which was expected as there is a delay between Z ring formation and recruitment of the late division proteins such as FtsI and FtsN. In the case of HADA incorporation, we found that a band of HADA was present at 50-70% of the ZapA-mcherry ring before the induction of the FtsW mutants. This is again expected. However, very few HADA bands were observed at the ZapA-mcherry rings after induction of the FtsW mutants. These results were included as S5_Fig and S6_Fig in the revised manuscript (Line270-300).

2. Do the authors believe that the presented colocalization experiments can definitively prove that the divisome complex is fully assembled in the context of the FtsW mutants? If not, conclusions about the effect of mutations on divisome assembly should be toned down.

**Response**

We believe that the co-localization of ZapA with late divisome proteins such as FtsI and FtsN as a good indication for the assembly of the divisome core (meaning a complex containing all the essential divisome proteins). However, it is likely not correct to say it represents a fully assembled divisome because many accessory division proteins localize to the division site after septal PG synthesis is initiated. We tried to be as accurate as possible with our wording in our revised manuscript.

3. In figure 9, the authors use co-expression and a pulldown assay via Nickel-NTA resin to show that FtsW and PBP3 retain a stable complex, even in the context of nonfunctional mutations. However, by eye, the ratios of FtsW-to-PBP3 appear to be somewhat variable. A quantification of the populations of the complex vs dissociated components by size-exclusion chromatography would provide a more rigorous analysis of the binding interaction. Additionally, the pulldown experiment should include as a negative control a lane for the FtsI expressed in the absence of FtsW. Many proteins (particularly penicillin-binding proteins) can stick non-specifically to NTA resin. A related minor point is that the description of the pull-down in the main text is very brief, and providing slightly more detail here would be helpful.

**Response**

We thank the reviewer for the suggestions.

Some variation in the ratio between FtsW and PBP3 can be observed even between different purifications of the WT proteins due to the limited affinity between the two proteins of the complex. During the optimisation of the purification of the complex, we chose a washing condition that is a compromise between the stability of the complex and the quality of the purification. From our previous experience with these proteins, gel filtration does not improve the ratio of the two proteins, probably because of detergent micelles.

We have now added negative controls for the purification of HisFtsW-FtsI (PBP3) in **S9_Fig**. The results from the co-expression of HisFtsI(PBP3)-FtsW or HisPBP3-FtsW_HA_ (FtsW_HA_ has a sort peptide (9 aa) insertion between residues E293 and A294 in the large loop between TM7 and TM 8) show that in contrast to FtsW, the FtsW_HA_ mutant, which does not bind to FtsI (PBP3), is not retained by Nickel column and can be detected in the flow through using anti-HA antibodies (**S9A-B_Fig**). Similarly, when untagged FtsI (PBP3) was co-expressed with HisFtsN and purified under the same condition as HisFtsW-PBP3, HisFtsN was present in the elution fraction but FtsI (PBP3) was found in the flow through (**S9C_Fig**) showing that FtsI (PBP3) does not bind non-specifically to Nickel column and that co-elution of FtsI (PBP3) with FtsW and the mutants is mediated by its interaction with FtsW (line 337-347).

More details for the purification of HisFtsW-PBP3 complex are provided in the main text and also the materials and method section of the revised manuscript.

4. The authors argue that their dominant-negative mutants are not defective in the “activation” step of the catalysis, and present three arguments to support their claims. However, counter arguments can be made for each case.

a. A well-characterized mutant (M269K) that is defective in activation is less toxic than the newly isolated mutants. However, the relative toxicity of the variants could be explained by differences in the degree of activation (or expression, degree of foldedness, etc.) rather than differences in the mechanism of disruption.

**Response**

We thank the reviewer for this comment. Based on our experience with FtsW, mutants partially defective in activation are able to complement when overexpressed and are not very toxic. However, the mutants isolated in this study are completely dead in the complementation test and are very toxic. Since most of the dominant-negative mutants are more toxic than the M269K activation mutant and as toxic as or even more toxic than the D297A mutant, which is a putative catalytic mutant based on characterization of similar FtsW mutants from other organisms, we believe that they are similar to D297A or represent a different class of mutant rather than activation defective mutants. However, we agree with the reviewer that we cannot exclude the possibility that some of the mutants may be also involved in activation. We tried to be as cautious as possible with our wordings in the revised manuscript.

b. The position of the mutants (some of them are in the “cavity” rather than facing the extracellular interface of FtsW) is not consistent with disruption of FtsW-PBP3 interactions, as reported previously for RodA and its cognate enzymatic partner PBP2. However, the fact that known residues involved in allostery between these proteins tend to cluster in one location does not exclude the possibility that other regulatory residues are yet to be identified. Another important consideration is that a number of identified mutants do map to the extracytoplasmic loops of FtsW in the AlphaFold model of the protein. Given that these loops are likely to be dynamic, it is difficult to determine a priori what their orientation and function would be in the PBP3-bound state of FtsW - facing the inner cavity (presumably, catalytic), or at the interface with PBP3 (presumably, critical for binding of the two components), or perhaps both.

**Response**

We agree with the reviewer that we could not exclude the possibility that some of the residues might be also involved in interaction with FtsI because they do locate at positions that could be part of the interaction interface between FtsW and FtsI. However, based on our characterization of the mutants, we think the most likely explanation is that they are involved in catalysis instead of activation. Ultimately, thorough biochemical analysis of these mutants and structures of FtsW-FtsI or other SEDS-bPBP complexes with and without ligands and at different states are probably required to reveal the definite defect of these mutants. We added these alternative possibilities in the discussion section.

c. Suppression of M269K defects by the E289G mutation in FtsW may be specific to the interaction of these residues and may not apply globally to other residues involved in activation.

**Response**

Thanks for this comment. Although it is possible, however, we think it is unlikely the suppression of M269K by E289G is specific between these two residues. The E289G mutation functions as a general suppressor of FtsW mutants defective in activation (M269K, and A270T) and also many FtsI mutants defective in activating septal PG synthesis (Li Y, et al. *PLoS Genet*. 2021 Apr 15;17(4):e1009366). It also suppresses an FtsW mutant (R172D, located in the cytoplasm) which is supposed to be unable to receive the activation signal from FtsA (Park et al. 2021 mBio). E289G appears to lock the protein in the active form, although how it works remains to be determined.

5. In figure 10, the authors measure lipid II binding using a fluorescence anisotropy binding assay. The intrinsic properties of the lipid II molecule as well as detergent solubilized proteins make them prone to non-specific interactions. Thus, it is difficult to verify the results of the lipid II binding assay in the absence of internal negative controls. If there are known mutations that disrupt lipid II binding in FtsW or lipid II analogs with similar chemical properties, the authors can use either a mutant protein or a non-interacting lipid analog as a negative control. In the absence of these tools, the authors should include a non-binding membrane protein control to validate their approach.

**Response**

We appreciate the reviewer’s suggestion. However, we are not aware of any lipid II binding mutants of FtsW. The interaction of SEDS proteins with lipid II is well established and this binding experiment is consistent with the observed in vivo activity of FtsW. Nonspecific binding often shows erratic behavior which do not observe in our experiments with different proteins known to bind to lipid II. The full-length membrane protein FtsN solubilized in detergent was used as negative control and it does not show significant interaction with lipid II probe, indicating that the observed interaction between FtsW-PBP3 and lipid II is specific. The data are published in Boes et al. Sci Rep 2020 10(1) : 6280. See figure below (for review only). Also, in the same paper it was shown that the antibiotics nisin and ramoplanin, which bind the pyrophosphate portion of lipid II prevent binding of lipid II to FtsW or PBP1b, indicating that the binding is specific.

 

6. As mentioned above, the fact that purified WT FtsW-PBP3 has no detectable polymerization activity is highly problematic and calls into question the results of other in vitro assays (particularly, the lipid II binding assay). Additionally, since the main claim of the manuscript is that the identified mutants are deficient in polymerization, it is critical that the authors are able to probe catalytic activity directly.

**Response**

We agree with the reviewer 100% that it is critical to probe the catalytic activity of these mutants to confirm that they are defective in catalysis. However, the *in vitro* polymerase activity of FtsW-PBP3 from *E. coli* could not be detected both in our hands and also by Marmont and Bernhardt (Proc Natl Acad Sci U S A. 2020, 117(38):23879-23885). As a result, we could only characterize the binding property of FtsW with lipid II by fluorescence anisotropy binding assay. This is also why we used “the potential active site” in our title of the manuscript.

**Minor issues:**
1. In Figures 5-8, the mutants used in the assays were not the same. Why were different mutants included in Figure 5 than in 6-8? Of particular note is the lack of the D297A mutant, which is a control used in most other assays.

**Response**

We had carried out the experiments for all the mutants (but at different time points during the study), they behaved similarly. When we generated the figures we tried to use mutants from the same batch and D297A was not in the same batch as the other mutants when it was tested so it was not included in Figure 5.

2. In the main text, please include a description of ZapA-mCherry and its use as a proxy for the presence of the Z-ring

**Response**

This was mentioned in line 274 in the original manuscript “… with ZapA-mcherry as a proxy for the Z ring.” However, this may be oversimplified and not sufficient for readers outside of the field. So we changed this sentence to “To rule out this possibility, we checked the localization of FtsI and FtsN, which are markers for mature divisomes, in cells overexpressing the dominant-negative FtsW variants. The test was carried out in a strain expressing ZapA-mcherry at its native locus, which interacts directly with FtsZ and has been widely used as a proxy for the Z ring.”

3. In Figure 3, a legend for the colors used to indicate residues identified by various methods should be included within the figure to increase clarity.

**Response**

Thanks for the suggestion, it was included as suggested.

4. The phrase “This cavity is ~15 Å wide by ~30 Å tall and is large enough to accommodate a lipid II molecule” is taken directly from another paper and should be re-written.

**Response**

Thanks for pointing this out, it was reformatted in the revised manuscript as “Since this cavity is large enough to bind a lipid II molecule (10), it has been suggested that this cavity is a substrate entry or exit site for lipid II.”

5. Typo on line 370 – “shown up” should be “show up”

**Response**

Changed as suggested.

6. The AlphaFold2 model is described as a “homology model” which isn’t quite correct. AlphaFold2 incorporates data from evolutionary sequence analysis, protein folding properties learned from the PDB, and homologous structures, resulting in models that are different from (and more accurate than) conventional homology models.

**Response**

Thanks for correcting our misconception, “homology model” was removed from the revised manuscript.

**Reviewer #2:** This paper from Li an co-workers investigates the function of the SEDS protein FtsW responsible for building the peptidoglycan (PG) cell wall at the division site. In the last several years, it has become clear that complexes formed by PG polymerases of the SEDS family and class B PBPs (bPBPs) that crosslink PG are the essential PG synthases of the elongation and division machineries of bacteria. The SEDS protein RodA of the elongation machinery has been characterized structurally and subjected to mutagenesis to identify residues required for its activity. As a result, regions of the protein thought to form the active site have been identified.

In this paper, the authors use a clever genetic screen to identity dominant-negative mutants of FtsW, the cell division counterpart of RodA. When mapped onto a model of the FtsW structure, the altered residues in the isolated mutants cluster around what looks to be the active site pocket of FtsW that is analogous to that previously described for RodA. Cell biological and biochemical studies of the altered proteins support the conclusion that these altered FtsW proteins are indeed defective for catalysis.

Overall, the paper is well written, and the results are clearly presented. I also agree with the conclusions. However, the paper could be improved by the addition of some quantitation of the protein localization experiments and the addition of some controls for the biochemical assays.

**Response**

We thank reviewer #2 for his/her kind words on the manuscript and constructive suggestions. In the revised manuscript, we quantified the co-localization of GFP-FtsI and GFP-FtsN with ZapA-mcherry as well as the co-localization of HADA labelling with ZapA-GFP. The results support the representative images in Figure 6, 7, and 8 and were included as S5_Fig and S6_Fig. We also included control experiments for the co-purification of HisFtsW-PBP3, which shows that PBP3 does not bind non-specifically to Ni-NTA column and that co-elution of PBP3 with FtsW and the mutants is mediated by its interaction with FtsW. These results were included as S9_Fig.

**Major Points:**

1) The biochemical experiments in Figure 9 lacks a control to show that FtsI(PBP3) purification is specific for cell producing tagged FtsW or its derivatives. At a minimum, I would like to see an untagged FtsW control showing that the proteins isolated via NiNTA chromatography require the tag. Even better would be the addition of control FtsW mutants with amino acid changes that are known to disrupt the interaction with PBP3 (or alternatively PBP3 mutants known to be defective in interacting with FtsW).

**Response**

We thank the reviewer for this suggestion.

- Negative controls for the purification of HisFtsW-PBP3 are provided in **S9_Fig**. We co-expressed HisFtsI (PBP3)-FtsW or HisPBP3-FtsW_HA_ (FtsW_HA_ has a sort peptide (9 aa) insertion in between residue E293 and A294 in the large loop between TM7 and TM 8, which prevents interaction with PBP3). The results show that in contrast to FtsW, the FtsW_HA_ mutant, which does not bind to PBP3, is not retained on the Nickel column and is detected in the flow through using anti-HA antibodies (**S9A-B_Fig**). Similarly, when untagged PBP3 was co-expressed with HisFtsN and purified under the same condition as HisFtsW-PBP3, FtsN was present in the elution fraction but PBP3 was found in the flow through (**Fig.** **S9C**) showing that PBP3 does not bind non-specifically to Nickel column and that co-elution of PBP3 with FtsW and the mutants is mediated by its interaction with FtsW (Line 337-347).

2) As in Figure 9, the biochemical experiments in Figure 10 lack a control for the specificity of the observed signal. Lipid II is hydrophobic and has the propensity to non-specifically associate with other hydrophobic entities, such as membrane proteins in detergent. Therefore, the Lipid II binding experiments require a controls - detergent with no protein added, and detergent micells with a control polytopic membrane protein not expected to bind lipid II. Without these controls, the anisotropy data is difficult to interpret and could be the result of non-specific interactions with lipid II.

**Response**

The interaction of SEDS proteins with lipid II is well established and this binding experiment is consistent with the observed in vivo activity of FtsW. Nonspecific binding often shows erratic behavior which do not observe in our experiments with different proteins known to bind to lipid II. In our binding experiments blank samples without protein (same buffer and detergent condition used for the protein) were also included for estimation.

The full-length membrane protein FtsN solubilized in detergent was used as a negative control and it did not show significant interaction with lipid II probe, indicating that the observed interaction between FtsW-PBP3 and lipid II is specific. The data are published in Boes et al. Sci Rep 2020 10(1) : 6280. See figure below (for review only). Also, in the same paper it was shown that the antibiotics nisin and ramoplanin, which bind the pyrophosphate portion of lipid II prevent binding of lipid II to FtsW or PBP1b, indicating that the binding is specific.

3) The micrographs characterizing localization of the FtsW mutants and other divisome proteins or septal PG synthesis when these mutants are produced require some quantification in addition to the micrographs shown. For example, the number of rings observed per cell length should be reported for each experiment, or some equivalent measurement to compare the different effects of the mutants on divisome formation and activity.

Response

We thank the reviewer for this suggestion. We had quantified the co-localization experiments presented in Figure 6, 7 and 8. The results are consistent with the representative images and included as S5_Fig and S6_Fig in the revised manuscript (Line 270-300). Also see our response to reviewer #1’s major issue 1.

(Before induction of the FtsW variants, we found that about 70% of the ZapA-mcherry rings were associated with GFP-FtsI or GFP-FtsN rings. After induction of the FtsW variants, the frequency of co-localization was increased, but there were also a few ZapA-mcherry rings without a GFP-FtsI or GFP-FtsN rings, which was expected as there was a delay between Z ring formation and recruitment of the late division proteins such as FtsI and FtsN. In the case of HADA incorporation, we found that a band of HADA was presented at 50-70% of the ZapA-mcherry ring before the induction of the FtsW mutants. However, very few HADA bands were observed at the ZapA-mcherry rings after induction of the FtsW mutants. These results were included as S5_Fig and S6_Fig in the revised manuscript.)
